# Séro-épidémiologie du VIH et des hépatites virales B et C chez les tradipraticiens dans le district de santé de Dschang: distribution et facteurs favorisants

**DOI:** 10.11604/pamj.2025.52.192.49468

**Published:** 2025-12-31

**Authors:** Jovita Dolly Eteuh Atontsa, Cavin Epie Bekolo, Djerry Dunhill Nzapze, Cedric Darel Wambo, Aurelle Christelle Noula, Alexis Saamene, Charles Kouanfack

**Affiliations:** 1 Global Research Agency Association, Dschang, Cameroon,; 2 Direction de Lutte contre la Maladie, les Épidémies et les Pandémies, Ministère de la Santé Publique, Yaoundé, Cameroun,; 3 Faculty of Medicine and Pharmaceutical Sciences, University of Dschang, Dschang, Cameroon,; 4 Hôpital Régional Annexe de Dschang, Dschang, Cameroun,; 5 Centre de Coordination des Opérations d'Urgences de Santé Publique, Ministère de la Santé Publique, Yaoundé, Cameroun

**Keywords:** VIH, VHB, VHC, tradipraticiens, district de santé de Dschang, HIV, HBV, HCV, traditional healers, Dschang health district

## Abstract

**Introduction:**

au Cameroun, les tradipraticiens (TR) sont fortement exposés aux infections transmissibles par le sang en raison de leurs pratiques professionnelles. Cependant, il existe un manque crucial de données concernant la séro-épidémiologie du virus de l'immunodéficience humaine (VIH), ainsi que des virus des hépatites B (VHB) et C (VHC) dans cette population. L'objectif de cette étude était de décrire le profil épidémiologique de ces infections chez les TR.

**Méthodes:**

il s'agit d'une étude transversale réalisée dans le district de santé de Dschang, entre février et avril 2024. Le protocole comprenait deux volets: un descriptif, pour établir les séroprévalences des infections étudiées, et un analytique, pour identifier les facteurs associés à ces séroprévalences. Conformément aux critères de sélection, tous les participants ont été recrutés de manière exhaustive. Les données ont été recueillies au sein de la communauté à l'aide d'un questionnaire standardisé administré en personne. Des statistiques descriptives ont été utilisées, suivies du calcul des Odds Ratios (OR) pour identifier les facteurs associés à la séroprévalence, avec un seuil de signification fixé à p < 0,05 et un intervalle de confiance (IC) à 95%.

**Résultats:**

parmi les 132 praticiens contactés, 114 ont été inclus dans l'étude. Les tradipraticiens ont présenté une séroprévalence de 9,4% (IC: [4,2-16,6]) pour le VIH, 2,1% (IC: [0-5,1]) pour le VHB et 3,1% (IC: [0-7,2]) pour le VHC. La majorité des participants (58,8%) avait un niveau de connaissance jugé faible. Globalement, les attitudes étaient positives, et la pratique la plus courante était la scarification (90,4%). Le seul facteur significativement associé à la présence d'infections était la pratique des autopsies traditionnelles (OR = 17,4 [1,54-198], p = 0,02).

**Conclusion:**

la prévalence du VIH chez les tradipraticiens est notablement élevée. Il est donc essentiel de leur fournir des équipements de protection individuelle et d'organiser des formations ciblées sur les infections transmissibles par le sang afin de réduire les risques de transmission.

## Abstract (en)

**Introduction:**

in Cameroon, traditional healers (THs) are highly exposed to bloodborne infections due to their professional practices. However, there is a critical lack of data regarding the sero-epidemiology of the Human Immunodeficiency Virus (HIV), as well as Hepatitis B (HBV) and Hepatitis C (HCV) among this population. The objective of this study was to describe the epidemiological profile of these infections among THs.

**Methods:**

this was a cross-sectional study conducted in the Dschang Health District between February and April 2024. The protocol included two components: a descriptive analysis to determine the seroprevalence of the studied infections, and an analytical component to identify contributing factors. According to our selection criteria, all participants were exhaustively recruited. Data were collected within the community using a standardised questionnaire administered in person. Descriptive statistics were followed by the calculation of the Odds Ratio (OR) to identify factors associated with seroprevalence, with a significance threshold set at p < 0.05 and a 95% confidence interval (CI).

**Results:**

of the 132 practitioners contacted, 114 were included in the study. The traditional healers showed a seroprevalence of 9.4% (CI: [4.2-16.6]) for HIV, 2.1% (CI: [0-5.1]) for HBV, and 3.1% (CI: [0-7.2]) for HCV. The majority of participants (58.8%) had a low level of knowledge. Overall, attitudes were positive, and the most commonly reported practice was scarification (90.4%). The only factor significantly associated with infection was the practice of traditional autopsies (OR = 17.4 [1.54-198], p = 0.02).

**Conclusion:**

the prevalence of HIV among traditional healers is notably high. Therefore, it is crucial to provide them with personal protective equipment and ensure they receive training on bloodborne infections to help reduce transmission risks.

## Introduction

Le virus de l'immunodéficience humaine, selon l'OMS, est une infection qui attaque le système immunitaire du corps, en particulier les globules blancs appelés cellules CD4, joue un rôle crucial dans la santé publique mondiale [1]. D'après les estimations de l'ONUSIDA, en 2022, 39 millions de personnes au niveau mondial étaient atteintes du VIH et 1,3 million de personnes nouvellement infectées [2]; le nombre total d'Africains vivant avec le VIH ou le sida était de 25,3 millions. Au Cameroun, selon les données de CAMPHIA 2017 et de l'EDS 2018 la prévalence du VIH chez les adultes âgés de 15 à 49 ans était respectivement de 3,4% et 2,7. En ce qui concerne les hépatites virales B et C, qui sont respectivement, un virus à ADN à tropisme hépatique, provoquant une nécrose et une inflammation des cellules hépatiques et une infection virale qui affecte le foie et qui peut engendrer une maladie aiguë et une maladie chronique, d'après l'OMS, plus de 91 millions de personnes en Afrique souffrent de l'hépatite B ou de l'hépatite C, qui sont les souches les plus fatales du virus, ce qui démontre que cette maladie reste une menace majeure pour la santé publique [3]; la prévalence de l'infection par le VHB est la plus élevée en Afrique et en Asie [4]; au Cameroun, selon les données de Gavi de 2022, la prévalence de l'hépatite virale C au Cameroun était de 1,3% contre 8,3% pour l'hépatite virale B.

Le personnel médical est souvent victime de piqûres, de coupures ou d'exposition de muqueuses à des matériaux potentiellement infectieux, ce qui peut entraîner une infection par le VIH, le virus de l'hépatite B et celui de l'hépatite C [5]. En ce qui concerne les maladies transmissibles par le sang et les liquides biologiques, le personnel de santé est particulièrement exposé aux virus de l'immunodéficience humaine (VIH), au virus de l'hépatite B (VHB) et au virus de l'hépatite C (VHC) [5]. On sait que ce risque d'accident d'exposition au sang est étroitement lié à la nature du geste effectué [6]. En prenant en compte tout cela, nous constatons que de nombreuses recherches ont été menées à l'échelle mondiale sur la contamination des professionnels de la santé, hors les tradipraticiens au vu de leurs pratiques professionnelles tel que, la pratique des scarifications ainsi que les traitements de ces infections, ils constituent une population à haut risque de contracter une infection à VIH, VHB ou VHC ceci malgré le fait que ces derniers sont reconnus dans le système de santé camerounais comme étant des acteurs de la santé publique [7]. La littérature reste très pauvre en ce qui concerne la séroprévalence du VIH, VHB et VHC chez les tradipraticiens, ceci remarqué par l'absence de publications antérieures au Cameroun a ce sujet dans cette population particulière, c'est la raison pour laquelle nous voulons décrire le profil épidémiologique de ces infections chez les tradipraticiens, en se basant sur les l'hypothèses selon lesquelles les pratiques de ces dernières, des mauvaises habitudes et de mauvaises connaissances vis-à-vis de ces infections les exposées à ceux-ci.

Ainsi, cette étude a pour objectif de décrire le profil épidémiologique du VIH, du VHB et du VHC chez les tradipraticiens du district de santé de Dschang en 2024. Elle s'attache à déterminer les séroprévalences de ces infections, à évaluer les connaissances, attitudes et pratiques des tradipraticiens à leur égard, ainsi qu'à identifier les facteurs susceptibles de favoriser leur transmission au sein de cette population, dans le but d'avoir des données sur lesquelles nous pourrons nous aligner pour trouver des stratégies pertinentes pour diminuer la prévalence de ces infections non seulement dans cette population mais également dans la population générale.

## Méthodes

**Type d'étude:** il s'agissait d'une étude transversale, dont le volet descriptif permettait de décrire l'épidémiologie du VIH, du VHB et du VHC chez les tradipraticiens et le volet analytique d'identifier les facteurs favorisant l'apparition de ces infections dans cette population.

**Lieu d'étude:** l'étude a été menée dans le district de santé de Dschang qui est l'un des 20 districts de la région de l'Ouest, situé dans le département de la Menoua. Il compte 22 aires de santé, 89 FOSA dont 40 publiques et 49 privées; la structure de référence de ce district est l'hôpital régional annexe de Dschang qui possède une unité de prise en charge des PVVIH et qui prend également en charge les patients vivant avec l'hépatite B et C. La population du district est estimée à 243182 habitants en 2023 et s'étend sur une superficie de 726,6 Km^2^, et pour cette population, nous avons environ plus de 200 tradipraticiens et 50 ASC répartis dans les 22 aires de santé que compte le district. Il est limité: au nord par la commune de Nzong-zem; à l'est par la commune de Fokoue et au sud par la commune de Santchou. Ce district a été choisi par convenance et la collecte de données a été faite de manière exhaustive.

**Situation épidémiologique des infections:** au cours de l'année 2022, selon les données du *District Health Information Software* (DHIS), le district de santé de Dschang a comptabilisé: i) 1201 cas de personnes infectées par le VIH; ii) 143 nouveaux cas de personnes infectées par le VHB; iii) 82 nouveaux cas de personnes infectées par le VHC.

**Période et durée de l'étude:** notre étude s'est étendue sur une durée de huit mois (08) allant de novembre 2022 à juillet 2023 et la collecte des données s'est faite en communauté de février à avril 2022, période correspondant à la saison sèche pour éviter les menaces climatiques lors de la collecte de données.

**Population d'étude:** notre population source était constituée des tradipraticiens du district de santé de Dschang.

### Critères de sélection

**Critères d'éligibilité:** i) être tradipraticien ; ii) être dans le district de santé de Dschang depuis au moins 12 mois; iii) exercer le métier concerné depuis au moins 6 mois.

**Critères d'inclusion et d'exclusion:** i) volet descriptif: ont été inclus dans notre étude les tradipraticiens résidant et reconnus dans le district de santé de Dschang et ayant donné leur consentement éclairé. ii) volet analytique: ont été inclus comme: cas: les tradipraticiens ayant été séropositifs à au moins une de ces infections; non cas: les tradipraticiens ayant été séronégatifs à toutes ces infections. Ont été exclus des cas ou des non-cas tout participant ayant refusé d'effectuer les tests de diagnostic rapide du VIH, du VHB et du VHC.

**Echantillonnage et stratégies de recrutement de la population:** le recrutement de la population d'étude s'est fait à partir d'un échantillonnage exhaustif; les TR ont été sélectionnés dans le district de santé de Dschang, choisis par convenance. Nous avons contacté les tradipraticiens à travers la liste fournie par le président des TR du DS de Dschang. Après avoir contacté ces derniers, des rendez-vous ont été pris pour l'administration du questionnaire et les tests de dépistage.

**Biais potentiels de l'étude:** i) biais de sélection: échantillon pas représentatif, auto-sélection, refus; ii) biais d'information: déclaration biaisée, rappel, mesure, interprétation culturelle; iii) biais de confusion: contexte, expérience, environnement de travail.

**Procédure technique:** i) pré-counseling et prélèvement: avant le prélèvement, une séance de pré-counseling a été menée au domicile du participant, durant laquelle l'équipe s'est présentée, a expliqué l'objectif de la recherche, les infections ciblées (VIH, VHB, VHC), les méthodes de dépistage, ainsi que les options de traitement disponibles. Toutes les questions du participant ont été répondues, puis un consentement éclairé a été obtenu. Le prélèvement a ensuite été réalisé dans des conditions d'hygiène strictes : après désinfection du doigt, une goutte de sang a été recueillie à l'aide d'une lancette et déposée sur les bandelettes de test, avant de procéder à une compression au point de ponction. ii) réalisation des tests: pour assurer la qualité des tests utilisés lors de l'enquête, ceux-ci ont été préalablement vérifiés sur des personnes dont le statut sérologique pour le VIH, le VHB et le VHC était déjà connu. Les tests du VHB et du VHC ont été réalisés à l'aide de bandelettes *One Step Rapid Test*, fabriquées en 2023 par l'entreprise Hightop en Chine. iii) post-counseling et remise des résultats: après l'annonce des résultats, un accompagnement adapté est proposé. En cas de résultat positif, le participant est écouté et rassuré, une explication claire du résultat est donnée, les besoins immédiats sont évalués, et des conseils sur les stratégies thérapeutiques ainsi qu'un référencement vers un centre de prise en charge sont proposés. Le participant est également encouragé à en parler à une personne de confiance et à son/sa partenaire. En cas de résultat négatif, une vérification de la bonne compréhension est faite, suivie d'un échange sur les émotions et les stratégies de prévention envisagées. Un rappel des messages du pré-counseling est effectué, accompagné de recommandations préventives et d'informations utiles.

**Définition opérationnelle des termes:** tradipraticiens: est une personne qui n'a pas de formation médicale, mais est reconnue par la communauté dans laquelle il (elle) vit comme étant compétente pour fournir des soins de santé en utilisant des plantes, des animaux et des substances minérales et certaines autres méthodes basées sur sociale, culturelle et religieuse ainsi que les connaissances, les attitudes et les croyances qui prévalent dans la communauté en matière de bien-être physique, mental et social et de la causalité de la maladie et le handicap, et étant enregistré au niveau du district de santé et donc possédant une carte de tradipraticiens. Bonne connaissance: score de bonnes réponses égal ou supérieur à 50%. Mauvaises connaissances: score de bonnes réponses inférieur à 50%. Attitudes positives: score supérieur à 50%. Attitudes négatives: score inférieur à 50%.

**Collecte de données:** elle a débuté par la rencontre du président des TR du DS de Dschang. Les participants ont été contactés par téléphone afin de prendre un rendez-vous pour une rencontre en face à face. Ensuite, la collecte de données s'est faite grâce à un questionnaire semi-structuré sur papier administré en face à face, suivie de la réalisation des tests de dépistage du VIH, du VHB et du VHC.

**Gestion des données:** les données collectées ont été préalablement vérifiées au moment du reportage sur la fiche et au moment de les faire entrer dans le masque de saisie conçu avec Cs Pro version 7.7. La vérification des incohérences et des manquants a été effectuée, les données ont été traitées et puis exportées sur le logiciel *SPSS* version 23 pour analyse, tout ceci en respectant les aspects de confidentialité.

**Analyse des données:** les données ont été exportées de Cs Pro vers *SPSS* (*Statistical Package for Social Sciences*) V 23 pour analyse. Les séroprévalences des infections ont été calculées par le rapport des participants séropositifs à la totalité des participants inclus dans l'étude. Les niveaux de connaissances et d'attitudes ont été évalués à travers le calcul de scores grâce aux questions d'évaluation de connaissances et d'attitudes qui avaient été incluses dans le questionnaire. Les facteurs ont été identifiés à travers des analyses bivariées (OR brut et IC=95%) et multivariées (OR ajusté et IC=95%) effectuées sur ce logiciel. Les graphiques ont été réalisés à l'aide de Microsoft Office Excel 2016. La distribution normale des variables quantitatives a été recherchée avec le test de Komogorov-Smirnov qui a indiqué que la distribution ne suivait pas une loi normale. Les statistiques descriptives usuelles ont été utilisées pour décrire les caractéristiques des populations : effectifs et proportions pour les variables qualitatives et médianes et intervalles interquartiles pour les variables quantitatives; le seuil de significativité a été pris à une valeur de P inférieure à 0,05 et un taux d'erreur acceptable de 5%.

**Considération éthique:** une clairance éthique a été obtenue du comité régional d'éthique de l'Ouest le 27 mars 2024 (N°393/27/02/ 2024/CE/CRERSH-OU/VP), avant le début de la collecte. Les données ont été traitées de manière confidentielle, stockées sur un ordinateur sécurisé et partagées uniquement avec l'équipe d'étude. Les documents étaient codifiés pour préserver l'anonymat, et les informations seront exclusivement utilisées à des fins scientifiques.

## Résultats

**Figure 1:**
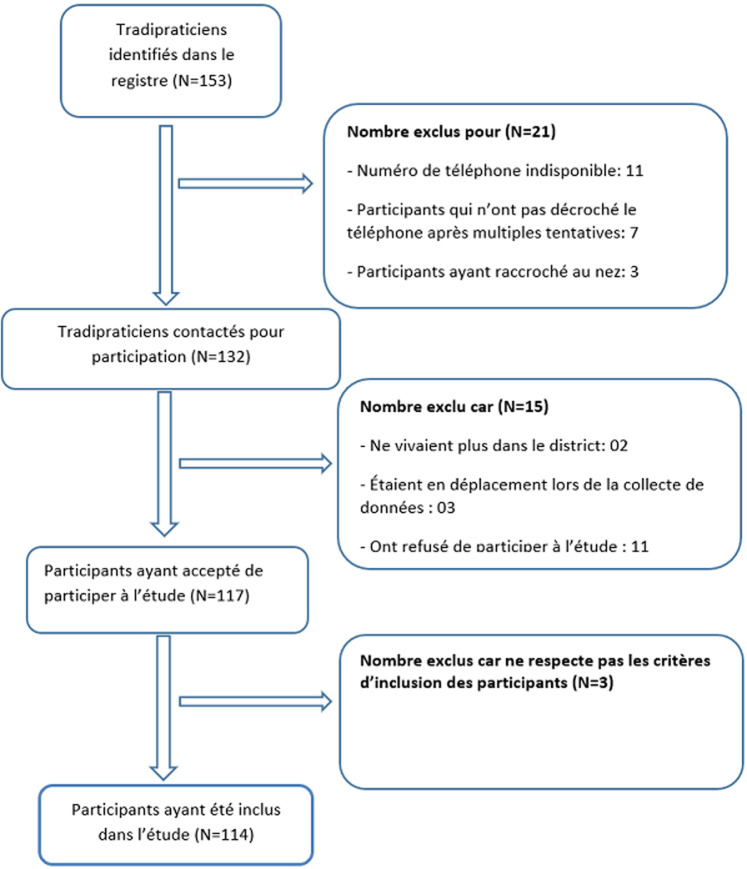
diagramme de flux des participants

**Diagramme de flux des participants:** parmi les 153 tradipraticiens enregistrés dans le district de santé de Dschang, seuls 132 ont pu être contactés et parmi eux, 117 ont accepté de participer à notre étude et seuls 114 ont été inclus dans l'étude, les autres ont été exclus de l'étude pour plusieurs raisons présentées dans le diagramme de flux des participants (Figure 1).

**Caractéristiques sociodémographiques des participants:** sur les 132 tradipraticiens éligibles, 114 ont accepté de participer à l'étude, soit un taux de participation de 91,2% contre 8,8% de refus. Les participants avaient un sex-ratio (hommes/femmes) de 1,03 et un âge médian de 43 ans (IQR: 39-60 ans), avec des extrêmes allant de 18 à 84 ans. Le mariage polygame était le type de conjugalité le plus fréquent (47,4%), et le niveau d'instruction dominant était le primaire (53,5%). L'aire de santé de Fiala Foreke était la plus représentée avec 30,7% des participants (Tableau 1).

**Tableau 1:** caractéristiques sociodémographiques des tradipraticiens dans le district de santé de Dschang en avril 2024

Variable	Modalités	Fréquence (N=114)	Pourcentage (%)
Age	18-30 ans	9	7,9
	31-50 ans	48	42,1
	51 ans et plus	57	50
Sexe	Masculin	58	50,9
	Féminin	56	49,1
Lieu de résidence	Urbain	58	50,9
	Rural	56	49,1
Statut matrimonial	Marié(e) polygame	44	38,6
	Marié(e) monogame	54	47,4
	Célibataire	10	8,8
	Veuf(ve)	6	5,3
Niveau scolaire	Non scolarisé	7	6,1
	Primaire	61	53,5
	Secondaire	43	37,1
	Supérieur	3	2,6
Expérience professionnelle	1-10 ans	35	30,7
	11- 21 ans	26	22,8
	22 et plus	53	46,5
Aire de santé	Baleveng	11	9,6
	Fiala foreke	35	30,7
	Fometa	19	16,7
	Fondenera	14	12,3
	Fkoue	-	-
	Ligang-foto	-	-
	Fontsa-touala	6	5,3
	fotsetsa	11	9,6
	Mbeng	1	0,9
	Maka	-	-
	Nkeuli	9	7,9
	Siteu	8	7

**Figure 2:**
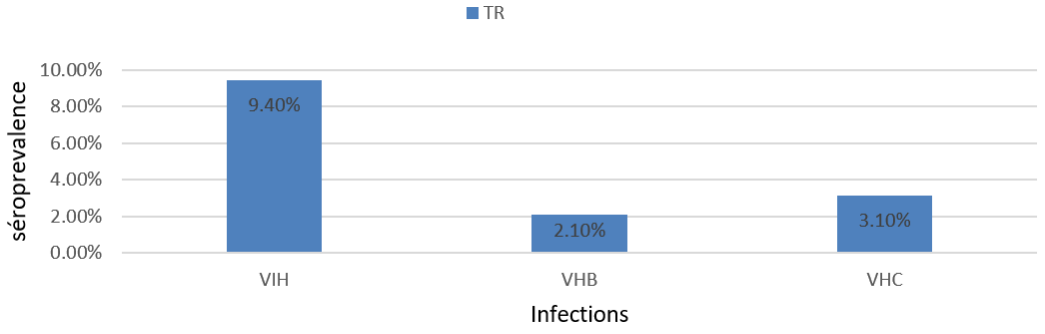
séroprévalence du VIH, VHB et VHC chez les tradipraticiens du district de santé de Dschang

**Distribution de la séroprévalence du VIH, du VHB et du VHC chez les tradipraticiens:** parmi les 114 tradipraticiens inclus dans l'étude, 96 (84,2%) ont accepté le test de dépistage du VIH et 97 (85,2%) ont consenti au dépistage du VHB et du VHC. La séroprévalence observée était de 9,4% pour le VIH (IC95%: 4,2-16,6), 2,1% pour le VHB (IC95%: 0-5,1) et 3,1% pour le VHC (IC95%: 0-7,2) (Figure 2).

**Évaluation des connaissances, attitudes et pratiques des tradipraticiens:** parmi les 114 tradipraticiens interrogés, 58,8% avaient de faibles connaissances sur les infections étudiées, tandis que 41,2% présentaient de bonnes connaissances. En revanche, 90,4% affichaient une attitude positive vis-à-vis de ces infections. Concernant les pratiques, les plus courantes et à risque étaient la scarification (90,4%), l'accouchement traditionnel (68,4%) et le traitement du VIH ou des hépatites (67,5%). Des pratiques moins fréquentes mais à risque incluaient la circoncision (11,4%) et les autopsies traditionnelles (16,7%) (Tableau 2).

**Tableau 2:** évaluation des pratiques des tradipraticiens dans le district de santé de Dschang en avril 2024

Variable	Modalités	Fréquence	Pourcentage (%)
Spécialiste dans le traitement d’une du VIH, VHB ou VHC	Oui	77	67,5
	Non	37	32,5
Pratiquer la scarification	Oui	103	90,4
	Non	11	9,6
Pratiquer les accouchements	Oui	78	68,4
	Non	36	31,6
Pratique de circoncision	Oui	13	11,4
	Non	101	88,6
Pratiquer les autopsies traditionnelles	Oui	21	18,4
	Non	93	81,6

### Facteurs favorisant la survenue du VIH, VHB et VHC chez les tradipraticiens

**Connaissances, attitudes et survenue du VIH, VHB et VHC chez les TR:** l'analyse bivariée a révélé une association significative entre un faible niveau de connaissance et la présence d'au moins une des infections (VIH, VHB ou VHC), avec une valeur de p = 0,029 et un odds ratio de 3,95 (IC à 95%: 1,07-14,53), indiquant que les tradipraticiens mal informés ont près de 4 fois plus de risque d'être infectés. En revanche, aucune association significative n'a été observée entre une attitude négative et la présence d'une infection (p = 0,563 ; OR = 0,736) (Tableau 3). Pratiques des TR et la survenue du VIH, VHB et VHC: l'analyse bivariée des pratiques des tradipraticiens révèle que deux pratiques sont significativement associées à la présence d'au moins une infection (VIH, VHB ou VHC): i) être spécialisé dans le traitement d'au moins une de ces infections (p = 0,029; OR = 3,95), ce qui multiplie par près de 4 le risque d'infection; ii) pratiquer des autopsies traditionnelles (p = 0,018; OR = 4,04), ce qui augmente également le risque d'infection par un facteur de 4,04; iii) en revanche, les pratiques comme les accouchements traditionnels, la circoncision et la scarification, bien que couramment exercées, ne montrent pas d'association statistiquement significative avec la présence d'infection (p > 0,05) (Tableau 4).

**Tableau 3:** relation entre le niveau de connaissance et d'attitudes des tradipraticiens sur la survenue d'une des infections dans le district de santé de Dschang en avril 2024

Variable	Modalités	Présence d’au moins une infection (%)	OR brute (IC à 95%)	Valeur P
Niveau de connaissances	Mauvais	11(78,6)	3,95 [1,07-14,53]	0,029
	Bon	3(21,4)		
Attitudes	Négative	6(42,8)	0,736[0,247-2,197]	0,563
	Positive	8(57,2)		

**Tableau 4:** relation entre les pratiques des TR et la présence d'une des infections dans le district de santé de Dschang en avril 2024

Variable	Modalités	Présence d’au moins une infection (%)	OR brute (IC à 95%)	Valeur P
Spécialiste dans le traitement du VIH, VHB ou VHC	Oui	11(78,6)	3,95 [1,07-14,53]	0,029
	Non	3(21,4)		
Pratiquer les accouchements	Oui	10(71,4)	1,273[0,366-4,423]	0,704
	Non	4(4,6)		
Pratiquer la circoncision	Oui	1(7,1)	0,721[0,83-6,257]	0,766
	Non	13(92,9)		
Pratiquer la scarification	Oui	13(92,9)	1,781(0,210-15,115)	0,592
	Non	1(7,1)		
Autopsie traditionnelle	Oui	6(42,9)	4,038[1,201-13,58]	0,018
	Non	8(57,1)		

**Régressions logistiques multiples:** dans l'analyse multivariée, la pratique des autopsies traditionnelles est le seul facteur significativement associé à la survenue des infections étudiées (VIH, VHB ou VHC), avec un odds ratio (OR) de 17,4, un intervalle de confiance à 95% allant de 1,545 à 198 et une valeur p de 0,021 (<0,05). Cela signifie que les tradipraticiens pratiquant des autopsies traditionnelles ont environ 17,4 fois plus de risque de contracter l'une de ces infections par rapport à ceux qui ne les pratiquent pas (Tableau 5).

**Tableau 5:** facteurs associés à la présence du VIH, VHB et VHC chez les tradipraticiens dans district de santé de Dschang en avril 2024

Univarié			Multivarié	
Variables	OR non ajusté (IC 95%)	Valeur p	OR ajusté (IC 95%)	Valeur p
Spécialiste dans le traitement du VIH, VHB et VHC	3,95 (1,07-14,53)	0,029	1,18 (0,167-8,446)	0,863
Autopsie traditionnelle	4,038 (1,201-13,58)	0,018	17,4 (1,545-198)	0,021
Attitudes négatives	9,603 (3,111-29,643)	0,0001	6,822 (0,435-107,07)	0,172
Mauvais niveau de connaissances	3,95 (1,07-14,53)	0,029	0,320[0,025-4,048)	0,7
Age de 31-50 ans	6,914 (0,862-55,431)	0,05	3,6	0,9

## Discussion

**Caractéristiques socio-démographiques des participants:** il ressort de notre étude que l'âge médian des tradipraticiens était de 43 ans, ce qui est superposable aux résultats d'Agbor *et al*. au Cameroun qui trouvaient un âge médian de 46 ans et à ceux de Bamouni *et al*. à Bobo-Dioulasso en 1994 qui rapportaient 51 ans d'âge moyen [8,9]. La tranche d'âge la plus représentée dans notre étude chez les TR était celle de 51 ans et plus (50%). C'est aussi le constat qu'avait fait Sangaré, qui rapportait dans son étude 62% de TR de cette tranche d'âge [10]. Ces âges avancés pourraient s'expliquer par la lenteur de l'acquisition du savoir et du savoir-faire pour l'exercice autonome du métier. Les TR passent plusieurs années d'apprentissage sous la houlette d'un autre TR avant de commencer à exercer seuls. Le sex-ratio chez les TR de notre échantillon était de 1,06. Ce chiffre est comparable à celui de Bamouni *et al*. à Bobo-Dioulasso en 1994, qui trouvait 1,07 [9]. Cette faible proportion de femmes qui semble être différente sur le terrain, pourrait s'expliquer par une faible inscription des femmes au district pour posséder une carte. Mais notre chiffre est différent de celui de Drissa Diallo *et al*. qui rapportaient plutôt 63,9% de femmes et 36,1% d'hommes (sexe ratio = 0,6) [11]. Ceci pourrait s'expliquer par le fait que l'étude de Drissa Diallo portait sur le traitement des femmes enceintes et que cette prise en charge est beaucoup plus assurée par les TR de sexe féminin que par les TR de sexe masculin. Nos résultats rapportent que 86% des TR étaient mariés et 8,8% étaient célibataires. Ces résultats sont comparables à ceux de Bamouni *et al*. qui trouvaient 82,1% de mariés et 5,3% de célibataires à Bobo-Dioulasso en 1994 [9].

Nos résultats rapportent que le nombre moyen d'années d'expérience était de 14,70 ans. Ce résultat est contraire à celui d'Agbor *et al*. au Cameroun (21 ans) [8]. Près de la moitié (44%) de notre échantillon avait un nombre d'années d'expérience compris entre 22 et plus. Ceci est contraire aux résultats de Toudji Bandje *et al*. au Togo en 2007 qui trouvaient 54% ayant entre 10 et 20 années d'expérience [12]. La notoriété s'acquiert avec l'ancienneté.

**Séroprévalence du VIH, VHB et VHC chez les tradipraticiens:** concernant notre objectif qui était de de déterminer la séroprévalence du VIH, VHB et VHC, il ressort de notre étude que, la séro- prévalence du VIH chez les tradipraticiens était de 9,4%, séroprévalence qui très élevé par rapport à celle de la population générale du Cameroun qui était de 2,7% en 2018 [13] cette différence pourrait être expliquer par le fait que les tradipraticiens ont un taux de contact élevé avec les personnes infecter par le VIH, l'étude réalisée par Agbor *et al*. au Cameroun montre que les tradipraticiens affirmaient recevoir au moins 7 personnes infectés par le VHI par semaine [8]. La séroprévalence du VHB et du VHC chez les tradipraticiens était respectivement de 2,1% et 3,1%, ces résultats sont contraires à ceux trouvés par Bigna *et al*. au Cameroun, qui trouvaient une séroprévalence du VHB et du VHC dans la population générale respectivement de 11,2% (IC à 95%: 9,7-12,8) et 6,5% (IC à 95%: 4,5-8,8) [14,15]. Ceci est probablement dû au fait que la taille de notre échantillon n'était pas assez grande et portait uniquement sur les tradipraticiens enregistrés au niveau du district et donc serait plus conscient que les autres tradipraticiens.

**Evaluation du niveau de connaissances, attitudes et pratiques des tradipraticiens:** en ce qui concerne notre objectif qui était d'évaluer le niveau de connaissance, attitudes et pratiques des TR, il en ressort que, 58,8% (67) des tradipraticiens avaient de mauvaises connaissances en ce qui concerne le VIH, VHB et le VHC ces résultats sont similaires à ceux publiés par kyambikwa bisangamo *et al*. en RDC qui rapportait 47,9% [12] de tradipraticiens ayant de mauvaises connaissances en ce qui concerne le VIH, ces taux de mauvaises connaissances pourraient être due au fait que la plupart des tradipraticiens qui ont participés à l'étude ont un bas niveau scolaire, plus de la moitié des tradipraticiens soit 53,3% avaient arrêté l'école au cycle primaire. Il en ressort également que, les attitudes des tradipraticiens en ce qui concerne le VIH, le VHB et le VHC sont en générale positive soit 90,4% des tradipraticiens avaient une attitude positive, ces résultats sont contraires à ceux publiés par Kyambikwa Bisangamo *et al*. en RDC qui rapportaient 4,2% d'attitudes positives aux VIH chez les tradipraticiens cette différence pourrait s'expliquer par le fait que les tradipraticiens de notre étude étaient tous enregistrés et possédait donc une carte qui leurs donnait la qualification de tradipraticiens et donc ces derniers sont plus conscients que ceux qualifié de charlatan [13].

Il en ressort également de notre étude que les pratiques les plus utilisées par les tradipraticiens étaient, la scarification (90,4%), les accouchements traditionnels (68,4%) et les traitements des infections étudiées (67,5%), en dehors de ces pratiques, d'autres pratiques telles que les autopsies traditionnelles (18,4%) et les circoncisions (11,4%) sont pratiquées par quelques-uns des tradipraticiens et ASC qui ont participé à notre étude. Ces pratiques sont compréhensibles au vu du fait que, bien que ces pratiques exposent ces derniers aux infections transmissibles par le sang, elles restent la particularité de la médecine traditionnelle.

**Facteurs favorisant la survenue du VIH, VHB et VHC chez les tradipraticiens:** plusieurs facteurs pouvant favoriser la présence du VIH, VHB et VHC chez les tradipraticiens ont été étudiés et il en ressort que, les mauvaises connaissances (p=0,024), les attitudes négatives (p=0,0001), le fait d'être spécialiste dans le traitement d'une des infections (p=0,04) et la pratique des autopsies traditionnelles (p=0,018) sont des facteurs qui favorisent la présence de ces infections dans ces populations. Mais après avoir effectué une analyse multivariée, nous avons constaté que le seul facteurs qui favorise réellement la survenue du VIH, VHB et VHC chez les tradipraticiens était la pratiques des autopsies traditionnelles (p=0,059), cela pourrait s'expliquer par le fait que lors de la pratique des autopsies traditionnelles par les tradipraticiens, ces derniers n'utilisent pas des équipements de protections individuels tel que les masques, les gants ou les gel hydroalcooliques, et également le matériels utilisés par ces derniers la plus part du temps ne sont pas des instruments qui ont été au pré-able désinfectés.

**Limites de l'étude:** concernant la validité externe de l'étude, notre échantillonnage n'était pas aléatoire et l'étude était limitée juste au district de Dschang, il convient donc d'émettre des réserves quant à la généralisation des résultats. Cependant, cette étude peut servir d'exemple pour être réalisée dans d'autres localités (étude multicentrique) pour avoir une vue d'ensemble de la distribution des séroprévalences du VIH, du VHB et du VHC chez les tradipraticiens. Des contraintes d'ordre temporel et financier nous ont également fait limiter la taille de l'échantillon de l'étude qui n'était plus aussi représentative du district de santé de Dschang, réduisant ainsi la validité externe de notre étude. Ceci peut s'expliquer par le fait que nous avons voulu travailler seulement avec les tradipraticiens reconnus par le district de santé. D'un point de vue technique d'enquête, la technique par entretien individuel a des limites. Le comportement et la personnalité de l'enquêteur peuvent avoir une influence sur les réponses. Le questionnaire a été administré à 100% par une personne de sexe féminin, ce qui pouvait influer sur la disposition à communiquer avec l'enquêteur. Le questionnaire a été administré dans la majeure partie des cas en langue française, ce qui pouvait entraîner des erreurs de compréhension du contenu des questions par les personnes interrogées ne maîtrisant pas bien la langue française. L'administration des questionnaires en exclusivité par nous-mêmes avait pour but de réduire ce biais de distorsion de contenu des questions, car nous comprenons le *yemba*, langue la plus parlée et comprise par les tradipraticiens du district. Sur le plan de l'analyse, nous avons fixé des critères de médecine moderne pour apprécier leurs connaissances alors que les conceptions des maladies et des symptômes sont différentes dans les deux domaines. Au vu de la petite taille de notre échantillon et au vu du fait que nous travaillons sur une population spécifique (les tradipraticiens), la puissance peut être limitée par le nombre restreint de participants, ce qui peut rendre les résultats moins fiables pour détecter des associations. Dans le cas de notre étude, cette puissance reste modérée, probablement autour de 50-60%, ce qui reste inférieur au standard de 80%. Malgré ces limites, notre étude garde quand même une importance, car elle permet d'avoir une vue de la séroprévalence de ces infections chez les tradipraticiens.

## Conclusion

Cette étude, menée auprès des tradipraticiens du district de santé de Dschang, a révélé une séroprévalence élevée du VIH. Les participants présentaient globalement de faibles connaissances sur le VIH, le VHB et le VHC, bien qu'ils aient manifesté une attitude positive envers ces infections. Plusieurs pratiques à risque, telles que la scarification, les accouchements traditionnels et les soins spécialisés du VIH ou des hépatites, ont été relevées. Toutefois, seule la pratique des autopsies traditionnelles s'est avérée significativement associée à la présence des infections étudiées. En ce qui concerne les perspectives, nous aimerions réaliser dans un futur proche une enquête plus étendue et mettre sur pied des stratégies ciblées nécessaires pour mieux contrôler ces infections et contribuer à l'atteinte des objectifs 90-90-90 de l'ONUSIDA.

**Recommandations:** au ministre de la Santé publique du Cameroun: i) cartographier ou identifier et développer les programmes d'encadrement des activités des tradipraticiens; ii) fournir aux tradipraticiens des kits comprenant des équipements de protection individuelle. Au district de santé: i) organiser un programme de formation sur les infections transmissibles par le sang chez les tradipraticiens afin d'améliorer leurs connaissances à ce sujet; ii) organiser un programme de formation des tradipraticiens sur l'usage des équipements de protection individuelle; iii) promouvoir les bonnes pratiques à travers des réglementations dans la médecine traditionnelle; iv) impliquer les tradipraticiens dans les différentes formations et suivre leurs activités. Aux tradipraticiens: i) mieux s'informer sur les infections transmissibles par le sang; ii) adhérer aux programmes de sensibilisation sur le VIH/HVB/HVC; iii) collaborer avec les structures sanitaires.

### Etat des connaissances sur le sujet

- L'étude menée par Celesti Kyambikwa Bisangama et ses collaborateurs a révélé que 47,9% des tradipraticiens avaient de mauvaises connaissances sur le VIH/SIDA, 45,1% des connaissances passables et seulement 7,0% de bonnes connaissances;
- En ce qui concerne les pratiques de contrôle des infections, cette même étude révèle que 43,7% des participants avaient de mauvaises pratiques, 52,1% des pratiques moyennes, et seulement 4,2% de bonnes pratiques;
- L'étude mentionnée a également démontré que l'analyse de régression linéaire multiple n'a pas décelé de variable personnelle ou démographique comme indicateur significatif du niveau de connaissance.

### Contribution de notre étude à la connaissance

- Notre étude permet de connaître la prévalence des infections à VIH, VHB et VHC chez les tradipraticiens du district de santé de Dschang;
- Elle renseigne également sur les pratiques à risque des tradipraticiens, pouvant constituer des facteurs favorisant la transmission de ces infections.

## Remerciements

Nous remercions sincèrement toutes les personnes qui, de près ou de loin, ont contribué à la réussite de ce travail. Nous souhaitons citer particulièrement: le chef de district de santé de Dschang, pour avoir facilité l'accès à la communauté; les chefs d'aires de santé, pour leur collaboration et leur soutien logistique; le chef des tradipraticiens du district, pour son implication et son soutien dans le contact avec les tradipraticiens; Aloliko, chef des tradipraticiens de l'aire de santé de Fiala Foréké, pour son accompagnement lors de la collecte des données; toutes les familles Atontsa, pour leur soutien moral et financier tout au long de ce travail; Tatanfack Sontia Emerson, pour son accompagnement moral et son appui financier; Dandem Philomène, pour sa contribution à la réédition du document.
